# Building SDN-Based Agricultural Vehicular Sensor Networks Based on Extended Open vSwitch

**DOI:** 10.3390/s16010108

**Published:** 2016-01-19

**Authors:** Tao Huang, Siyu Yan, Fan Yang, Tian Pan, Jiang Liu

**Affiliations:** 1 State Key Laboratory of Networking and Switching Technology, Beijing University of Posts and Telecommunications, Beijing 100876, China; yansiyu@bupt.edu.cn (S.Y.); yfan@bupt.edu.cn (F.Y.); pan@bupt.edu.cn (T.P.); liujiang@bupt.edu.cn (J.L.); 2 Science and Technology on Information Transmission and Dissemination in Communication Networks Laboratory, Shijiazhuang 050081, China

**Keywords:** SDN-based vehicular sensor networks in agriculture, connection state, self-learning, Open vSwitch, networking survivability

## Abstract

Software-defined vehicular sensor networks in agriculture, such as autonomous vehicle navigation based on wireless multi-sensor networks, can lead to more efficient precision agriculture. In SDN-based vehicle sensor networks, the data plane is simplified and becomes more efficient by introducing a centralized controller. However, in a wireless environment, the main controller node may leave the sensor network due to the dynamic topology change or the unstable wireless signal, leaving the rest of network devices without control, e.g., a sensor node as a switch may forward packets according to stale rules until the controller updates the flow table entries. To solve this problem, this paper proposes a novel SDN-based vehicular sensor networks architecture which can minimize the performance penalty of controller connection loss. We achieve this by designing a connection state detection and self-learning mechanism. We build prototypes based on extended Open vSwitch and Ryu. The experimental results show that the recovery time from controller connection loss is under 100 ms and it keeps rule updating in real time with a stable throughput. This architecture enhances the survivability and stability of SDN-based vehicular sensor networks in precision agriculture.

## 1. Introduction

Recently, mobile autonomous vehicles based on wireless multi-sensor network have been extensively applied in precision agriculture [1,2], e.g., in order for vehicles to have the capability of weeding, fertilization, yielding analysis and conducting other agriculture activities, wireless multi-sensor networks [3,4] can monitor the vehicle condition, real-time position and implement feature detection on a crop field which can be used for autonomous vehicle navigation [5]. The core feature of this vehicle sensor network is the existence of many dynamic mobile sensor nodes carried by vehicles, which means that the mobile nodes will join and disjoin this network anytime like a vehicle *ad hoc* network [6,7]. Therefore, it becomes important to ensure normal network communication between different sensor nodes.

On the other hand, the Software Defined Networking (SDN) technique has developed quickly [8]. SDN is a novel networking paradigm which decouples the control plane from the data plane. It makes packet processing and forwarding more flexible and programmable [9]. Along with SDN’s popularity, SDN-based wireless sensor networks, especially SDN-based vehicular sensor networks in the agriculture field [10,11,12], have stirred a lot of attention recently [13]. The use of SDN-based wireless sensor networks supports flexible data acquisition, and the effective monitoring and accuracy control of sensor vehicles for precision agriculture in a positive direction. In these SDN-based vehicle sensor networks, a mobile node is selected as a SDN controller and the other mobile nodes as switches to maintain only flow-based forwarding functions. The controller can use an open API to control other switches. Introducing SDN to vehicular sensor networks in agriculture could improve the management of the mobile network resources [14] and increase the production efficiency.

However, when a controller cannot communicate with switches, the performance of flow-based forwarding of the switches will be degraded in SDN-based vehicular sensor networks. This can happen in the following scenarios: (i) the vehicular sensor network topology dynamically changes [15] or a main controller leaves the network, in which case it has to take some time to select a new main controller. In this selection interval, most sensors’ communication in the data plane will be interrupted because switches don’t know how to forward newly arrived sensor data due to the loss of control [16]; (ii) while the topology of network has changed, switches will still use stale rules until the controller inserts/updates the flow table entries [12]. Therefore, in SDN-based vehicular sensor networks, these problems have a major impact on the performance of sensors and networks in agriculture. The survivability and communication stability call for further improvement.

Many attempts have already been carried out to resolve the above problems. For example, the optimization handoff mechanism [16,17], local agent [12] and OpenFlow’s double pipelines [18]. But there are still some challenging issues. First, how to detect the controller failure and perform failure recovery as soon as possible. Second, the mechanism shouldn’t limit the flexibility and benefit of SDN in vehicle sensor networks.

This paper proposes a new SDN-based vehicular sensor networks architecture approach for precision agriculture. This architecture can minimize the performance penalty of controller connection loss and maintain the scalability and flexibility of SDN at the same time.

Based on the idea of stateful processing and service implementation flexibility in the data plane [19], this architecture follows the standard OpenFlow philosophy and extends Open vSwitch to add a stateful match and self-learning service. Through those, it can be aware of different connection states, achieve smooth handoff and process incoming packets by different scenario states. Besides, we argue that the mobile vehicle sensor network’s topology may change when a controller communicates with switches which are unavailable at that time. In this case, it can be ensured that the rules are updated in time by different states to avoid using the old rules.

We develop connection-state processing and self-learning services on top of Open vSwitch (OVS) to present a prototype implementation. Through experiments, we show that when a controller disconnects, the recovery time is short enough to guarantee an uninterrupted communication in the network. Specifically, the main contributions of this paper are as follows:

- This new architecture implements a connection state service to detect in real-time the state of vehicle sensor networks, achieves smooth stateful matching according to different scenario states and extends self-learning as a new OpenFlow action to enhance packet-processing capability in the network data plane.
- This new architecture maintains the compatibility with OpenFlow, and the flexibility and scalability of SDN.
- We describe our prototype implementation on Open vSwitch and evaluate it using a controller failure recovery scenario. The experiment results show that it can achieve a stable throughput with a recovery time of less than 100 ms.

The rest of this paper is organized as follows: Section 2 presents the system architecture containing some function designs, followed by Section 3 in which the implementation of modules is described. These concepts are evaluated in Section 4. Some related works are discussed in Section 5. Finally, the paper concludes in Section 6.

## 2. System Architecture

This section gives the design details of the proposed system architecture. The system is designed with the following goals in mind. First, this architecture should maintain the compatibility with OpenFlow, and the flexibility and scalability of SDN. Second, the design can perform stateful matching according to different scenario states and service implementation flexibility in the data plane by the OpenFlow API. Third, this architecture can be easily implemented on existing OpenFlow switches, such as OVS [20].

### 2.1. System Architecture and Packet Processing Logic

**Figure 1:**
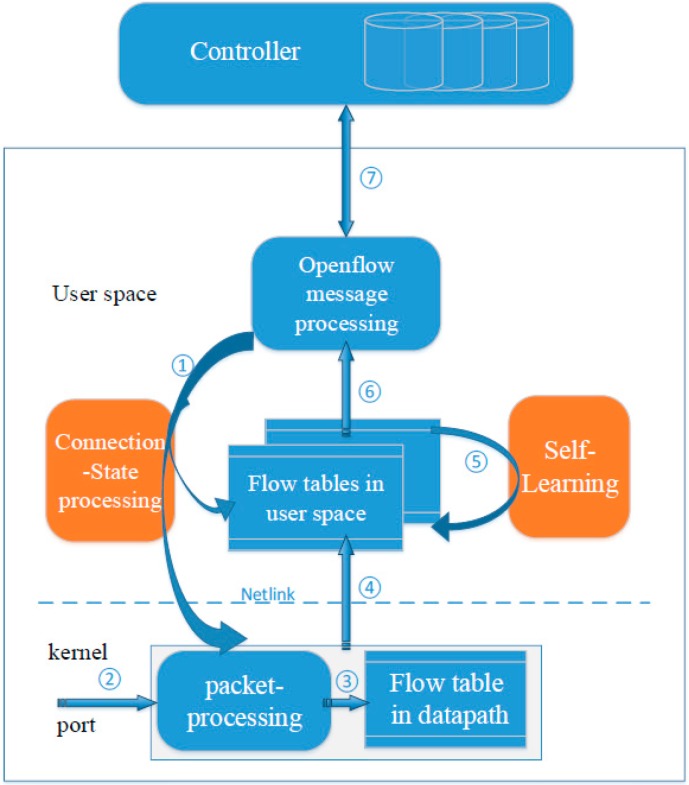
System architecture.

This proposed system architecture is mainly based on an extended OpenFlow switch. Figure 1 shows our proposed architecture. The blue parts are the existing main components in an OpenFlow switch, while the orange parts are developed by this paper.

The blue components include kernel datapath, user space flow table pipeline and OpenFlow message processing. Both the kernel’s datapath and user space contain flow tables. Packets can match a rule and execute the rule’s action. To speed up packet processing, most packets finish processing and forwarding in the kernel directly. OpenFlow message processing is in charge of receiving and sending OpenFlow messages with the controller. The extended parts include connection-state processing and the self-learning service. Connection-state can perform the connection state tracking, synchronization of connection states and stateful matching. Self-Learning can perform some packet-processing functions in the data plane by the action of OpenFlow and limit the need to send packets to the controller.

As shown in Figure 1, steps 1–7 show how the packets can be processed in this new architecture. The connection-state processing service can track the real-time connection state between a controller and a switch. Once the connection state changes, the connection state will be synchronously transmitted to the kernel and user space on time (see step 1 in Figure 1). When a packet of sensor data arrives at the switch’s ingress port, the kernel datapath will call the appropriate function to parse the packet header. Those fields parsed as well as the connection state together form a lookup key (see step 2 in Figure 1). Then the key is matched against the flow rules presented in the datapath flow tables (see step 3 in Figure 1). If there is no match, Netlink is used to send the packet as an Upcall message to the user space for lookup in flow table pipeline (see step 4 in Figure 1). If matching a rule in the user space, the packet will be processed according to the rule’s action, such as self-learning and the packet sent to the controller, *etc.* When the action is self-learning, it will parse the packet and insert/update a corresponding rule in the flow table (see step 5 in Figure 1). When the action is sending the packet to the controller, the OpenFlow message processing service will encapsulate the packet into a Packet-In message and send it to a controller (see step 6 in Figure 1). If connection to the switch goes well, the controller will receive and process the Packet–In message. Then the controller sends a Flow-Mod message to the switch and installs a new rule in the user space flow table (see step 7 in Figure 1). Finally, the actions of the new rule are executed on the packet and a new flow rule is inserted into the datapath so that the subsequent packets of the flow can be processed fast in the datapath.

### 2.2. Connection-State Processing Function

In the agriculture field, the vehicle sensor network is not stable. To achieve smooth handoff according to different scenario states when a packet matches rules, the connection-state processing function performs a stateful match by detecting the connection state between a switch and a controller and extending a new match field named connection-state.

At present, packets match non-stateful rules in an OpenFlow switch, because the match fields of rules only include L2–L4 fields extracted by packet headers and metadata fields like ImPort [21], which aren’t related to the state of the network. It is important to add a stateful matching field in the rules. Connection-state processing function realizes that a packet matches a specific rule in different connection states of a controller. To detect connection states and use them properly, there are two main steps: (i) connection state detection; (ii) connection state synchronization and encapsulation.

#### 2.2.1. Connection State Detection

The first step is to use a finite-state mechanism and transmission rate to get the real-time connection state. The connection state means how is the link between the SDN controller carried by a mobile node and other switches carried by other sensor nodes in the vehicle sensor network. Connection state can have different state values such as disconnection, connection, idle and busy.

For disconnection/connection state, it builds upon a finite state mechanism, whose states include ACTIVE, DILE, DISCONNECTED, *etc.* We define ACTIVE and IDLE as connected states. Other states such as DISCONNECTED are defined as disconnected states.

**Figure 2:**
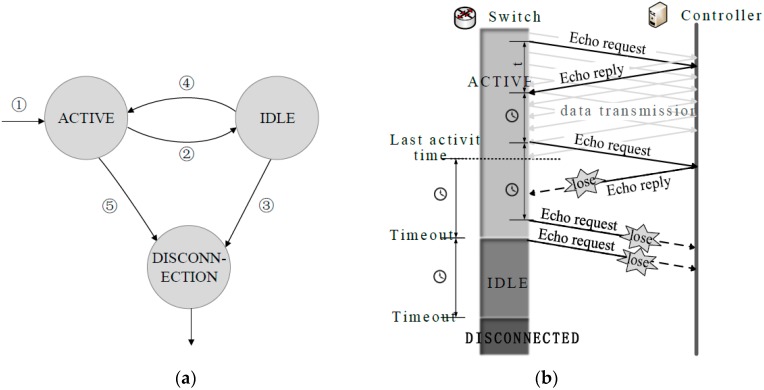
(**a**) State transition; (**b**) tracking connection state in the situation that the link is disconnected. In ACTIVE, because the switch can’t receive any messages or even echo reply messages from the controller, it moves into IDLE. Similarly, it finally moves from IDLE to DISCONNECTION.

As shown in Figure 2a, the proposed system can move from one state to another state according to certain conditions. To begin with, State machine enters ACTIVE when a switch with a controller builds a secure communication channel by version negotiation (see step 1 in Figure 2a). After that, the switch under ACTIVE or IDLE can use an echo message as a probe to detect the connection state. We take dynamic adjustment mechanism to change the interval for sending an echo message and the timeout period of different states, which are adjusted according to the last Round-Trip time (RTT) of the echo message. Timeout period means the switch can’t receive any OpenFlow messages from the controller during a period of time. After the timeout period, State machine will move from ACTIVE to IDLE (see step 2 in Figure 2a) or from IDLE to DISCONNECTION (see step 3 in Figure 2a). When in IDLE, state machine can return to ACTIVE if the switch receives an OpenFlow message and even an echo reply message from the controller during the timeout period (see step 4 in Figure 2a). When ACTIVE, the state machine can directly move into DISCONNECTION after the switch receives a socket disconnect request from the controller (see step 5 in Figure 2a), e.g., the vehicle carrying a controller initiates the steps to terminate the connection or SDN-Apps on a controller crash.

For vehicle sensor networks in the precision agricultural field, there are mainly two controller communication loss situations. Figure 2b shows the first situation where the link between a switch and a controller is disconnected, e.g., the mobile node serving as controller suddenly disjoins the network. In ACTIVE mode, the state machine will update the last activity time after the switch receives a message from the controller. If the switch can’t receive an OpenFlow message successfully from the last activity time to the timeout period, the switch enters IDLE and then sends out an echo request to the controller. In IDLE, the switch enters DISCONNECTED if the switch still can’t receive an OpenFlow message after a timeout period. Another situation is that a switch can enter DISCONNECTED directly after the switch receives a socket disconnect request from the controller, such as SDN-Apps on a controller crash or the successful handover of controller role. Note that the time of detection in the first situation is generally longer than in the second situation, but the experimental results show that proposed connection state detection mechanism can track the state as quickly as possible (refer to Section 4).

Idle/Busy state shows whether the wireless link between the controller and the switch is congested or not. Compared with disconnection/connection state, it is easy to detect idle/busy states by the transmission rate between the controller and the switch. The message queues in switches can show the current total number of OpenFlow messages which a switch receives and sends out, so the transmission rate between the controller and the switch can be calculated by the dynamic length of the message queues. The link is marked busy if the calculated transmission rate is larger than the set threshold, otherwise it’s idle.

#### 2.2.2. Connection State Synchronization and Encapsulation

**Figure 3:**
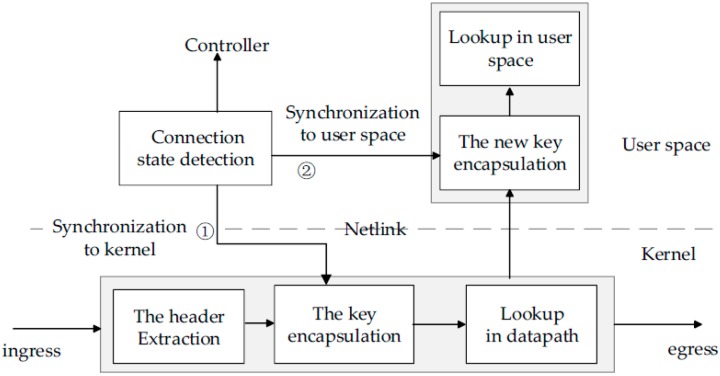
Synchronizing the real-time connection state to the kernel datapath and user space for encapsulating a lookup key. The key containing the connection state can match stateful rules.

The second step is that the connection state should synchronize to the user space and kernel datapath in the switch when the detected connection state changes. As shown in Figure 3, first, the connection state needs to be synchronously transmitted to the datapath by the user-kernel Netlink mechanism (see step 1 in Figure 3). When a packet arrives at a switch’s ingress port, a lookup key will be extracted from the header of the packet. At the same time, the connection state should be added into the lookup key-like Import field, which can make the key match stateful rules in the datapath. Second, the connection state value detected need be synchronously transmitted to the user space for an OpenFlow table lookup in the user space (see step 2 in Figure 3). In other words, a packet sent up to the user space should remove the old connection state and add a new connection state for forming a new lookup key in user space. This synchronization and the key encapsulation mechanisms ensure that a packet always uses a real-time state to match rules.

By the above design, the connection–state processing function can get the real-time connection state of a controller and synchronize it for packets to lookup in flow tables. Therefore, the state awareness and stateful match can be applied in many dynamically changing agricultural scenarios. For example, if the connection state between a sensor switch and controller is disconnection, packets can match those rules whose match fields include the disconnection state field. It can implement some services, such as self-learning function in the data plane when the controller is unavailable. Besides, if the connection state is busy, packets can be processed and forwarded into the bypass sensor node carrying a switch. If so, the bypass switch and another controller will process the packet, which achieves the load balance of controllers and improve the production efficiency in the agriculture field.

### 2.3. Self-Learning Function

The self-learning function mainly performs some services in the data plane, such as the L2-switch, which can be triggered flexibly by extended OpenFlow actions.

At present, all of OpenFlow actions only support the modification of the header of a packet and forwarding, but some simple services, such as L2/L3, NAT and firewall functions, can’t be implemented directly on the data plane, in which case the efficiency of the network sometimes is relatively low. By performing self-learning in an OpenFlow switch, packets can be processed on the data plane instead of being sent to the controller. This keeps the flexibility and benefit of SDN, because the controller can use the Openflow API, especially the action to call this service at an appropriate moment. In particular, a new rule generated by self-learning action is the same in spirit as the OpenFlow rules, so those rules can be inserted in the original flow tables with no difference.

The self-learning function combines both advantages of OpenFlow pipeline processing packets and L2 function of the traditional switch. First, if a packet executes a self-learning action, the source mac and ImPort field will be extracted from the header of the packet. According to this extracted information, it will generate a new rule whose “dst-mac” match field is the same as the source mac and OutPort action is the same as the ImPort. Second, it checks whether the user space flow tables contain a rule as same as the new rule. If not, the new rule can be directly inserted in flow tables. Otherwise, the old rules need be updated according to the new rule.

Self-learning function provides packet-processing service in the data plane, which can improve the network efficiency , so it can be applied in many SDN-based vehicle sensor network scenarios. If this function works, it decreases the number of packets sent to the controller and relieves the load of the controller. In some controller unavailable scenarios, the self-learning function can ensure that a switch processes packets and forwards them normally. It also ensures that SDN-based vehicle sensor networks work normally in agriculture scenarios.

### 2.4. An Example: Failure Recovery

We use an example to illustrate how the new architecture works. In a harsh agriculture environment, suppose the vehicle sensor network policy requires “data plane can maintain the basic communication when the controller is not available”. To implement this policy, the controller proactively inserts two rules in the first flow table: (match: connect_state = connection, actions = controller) and (match: connect_state = disconnection, actions: self-learning, go to table x). The self-learning rules are installed in flow table x.

When a new packet (src_mac = sm1, ImPort = p1) arrives at the ingress port of a switch, it will be delivered from the datapath in the kernel to the first flow table in the user space for lookup. The packet will get a current connectivity state and then use it to match against the rules. If the controller communication with this switch is available, the packet will match the first rule and be sent up to controller for further processing. Otherwise, the packet will match the second rule and execute two actions in sequence.

The first action is the self-learning action and should be executed on the packet. It will insert a new rule or update a corresponding rule in flow table x. If there is a rule whose dst_mac field is the same as sm1, the rule’s action should be updated to p1. Otherwise a new rule will be installed: (dst_mac = sm1, action = out p1). After that, this packet is set to table x for lookup. This packet may match against self-learning rules and be forwarded. Of course, there can be a miss-flow to process the mismatched packet specifically.

For a harsh agriculture environment, the above example can perform the failure recovery function. Communication in the vehicle sensor network will be uninterrupted even if the controller is not available.

## 3. Module Implementation

Open vSwitch [22] is an open source software switch that has become widely available in data centers, supporting diverse protocols such as OpenFlow [18]. Therefore, we select OVS as the basic platform to implement our new architecture in vehicle sensor networks. To enhance the scalability of OVS, this paper intends to make the functions of connection-state processing and self-learning modular. This section mainly describes how to implement the connection-state processing module and self-learning module, including extending OpenFlow match fields and actions, some structures and function definitions.

### 3.1. Connection-State Processing Module Implementation

**Figure 4:**
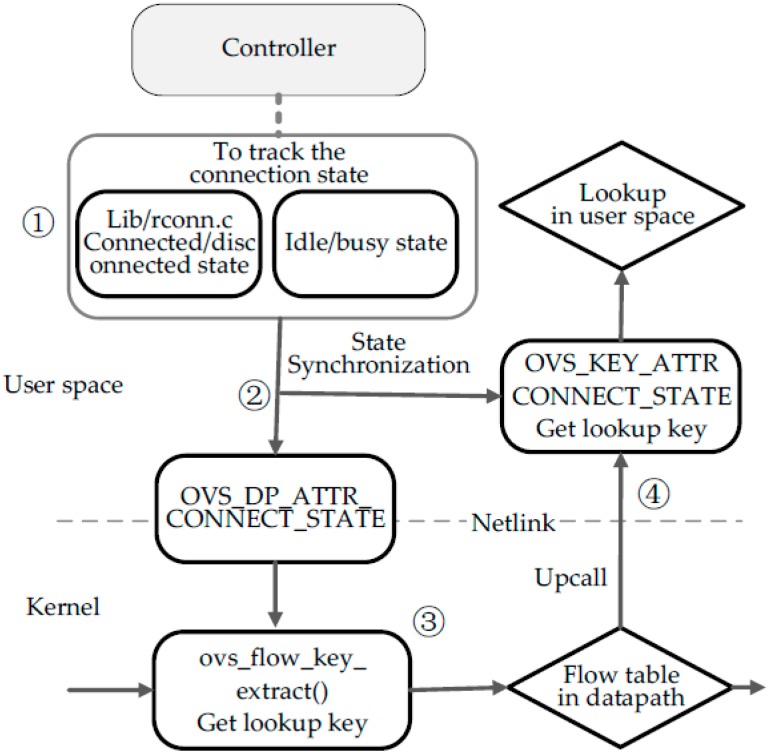
Connection-state processing module. Step 1 is to track the connection state. Step 2 is to transmit the state detected to the kernel and user space. Step 3 is to support a stateful match in the kernel. Step 4 is to support a stateful match in user space.

At present, the OpenFlow specification doesn’t contain a match field named connection state between a controller and switches. Therefore, we need to modify the Open vSwitch source code including the kernel and user space codes to implement a connection-state processing module. The implementation process refers to the flow chart shown in Figure 4.

Firstly, as shown in step 1 (see Figure 4), we need to extend OVS to detect the real-time connection state. Connection state detection builds upon a finite state mechanism and the processing functions of different states are defined in the file lib/rconn.c. In the function run_ACTIVE, an echo message is added to be a keep-alive probe, whose interval and different states’ timeout is adjusted dynamically according to the last round-trip time of echo messages. Besides, the last activity time of OpenFlow messages has to be recorded in function rcon_recv. Through the above mechanism, it will get real-time connected state value or disconnected state. When a packet matches a table-miss containing action Controller in the user OpenFlow pipeline, it will call ofproto_dpif_send_packet_in function and Packet-In message will be pushed in the transmit queues, so we detect the rate of enqueueing and then judge whether a state is idle or busy. The connection state enum is defined as follows (Algorithm 1):

**Algorithm 1**
enum connect_state{
 connection = 1;
 disconnection;
 busy;
 idle;
};


Secondly, as shown in step 2 (see Figure 4), the module will transfer the state value to both the user and kernel space for composing a lookup key in time. Connection state can be as a datapath attribute to be sent to the kernel datapath by General Netlink API. All of attributes supported by datapath are defined in the openvswitch.h file as mentioned below (Algorithm 2):

**Algorithm 2**
enum ovs_datapath_attr {
   OVS_DP_ATTR_UNSPEC,
   OVS_DP_ATTR_NAME,/* name of dp_ifindex netdev */   OVS_DP_ATTR_UPCALL_PID,/* Netlink PID to receive upcalls */   OVS_DP_ATTR_STATS,/* struct ovs_dp_stats */   OVS_DP_ATTR_MEGAFLOW_STATS,/* struct ovs_dp_megaflow_stats */   OVS_DP_ATTR_USER_FEATURES,/* OVS_DP_F_*/   __OVS_DP_ATTR_MAX
};


The new attributes are to be added the above enum definition like OVS_DP_ATTR_CONNECT_STATE. The attribute named OVS_DP_ATTR_CONNECT_STATE can transmit the connection state value into the kernel datapath. Then appropriate changes must be done to datapath/flow_netlink.c file.

Thirdly, as shown in step 3 (see Figure 4), we should add the match field into the lookup key structure to support a new match field named connection_state in datapath. In the flow.h file, there is a definition named sw_flow_key concerning the lookup key structure. Sw_flow_key structure has many match field attributes, including L2-L4 fields and some metadata fields, such as ImPort. Part of the structure is shown below. Therefore, the new match field named connection_state has to be added in the above structure. In this case, datapath can call ovs_flow_key_extract function to get lookup key. The lookup key contains not only some fields extracted from packets, but also connection_state from datapath attributes. Then this key is used to match against the flow rules in the kernel datapath.

Finally, as shown in step 4 (see Figure 4), the datapath will send up the lookup key and the packet to the user space for OpenFlow table lookup, if there is no match in the datapath. However, the connection state contained in lookup key is old, so we need to add a necessary ofproto-dpif-upcall.c function to update the connection state of the lookup key in user space. Besides, the new match field has some changes to metadata and others, so the appropriate changes must be done to the following files and structures, like pkt_metadata in lib/packet.h, flow and flow_metadata in lib/flow.h and lib/odp-util.c, *etc.*

### 3.2. Self-Learning Processing Module Implementation

Self-learning processing module implementation is mainly to extend OpenFlow actions, which can perform some services in the data plane, such as the L2-switch. All the action definitions are included in include/openflow directory. For example, in the openflow/openflow-1.3.h file, the structure named ofproto_action_type defines all types of actions. There are some action structure definitions. To support a new action, the new action type is to be added into enum ofp13_action_type like OFPAT13_SELF_LEARNING. Then an appropriate structure definition is to be defined like struct ofp13_action_self_learning in the same file (Algorithm 3):

**Algorithm 3**
struct ofp13_Action_self_learning {
   ovs_be16 type; /*OFPAT11_SELF_LEARNING. */
   ovs_be16 len; /* Length is 8. */
   uint8_t pad [4];
};

All the actions are supported by OpenFlow users pace and some actions extended by OVS are defined in the file ofp-actions.h. All the necessary structures used by these actions are also defined in the same file. The macro which defines actions is as follows (Algorithm 4):

**Algorithm 4**#define OFPACTS
DEFINE_OFPACT(OUTPUT, ofpact_output, ofpact)
DEFINE_OFPACT(GROUP,ofpact_group,ofpact)
DEFINE_OFPACT(CONTROLLER,ofpact_controller, ofpact) 
DEFINE_OFPACT(ENQUEUE, ofpact_enqueue, ofpact)

For self-learning action, we need to add the new action to the macro like DEFINE_OFPACT (SELF_LEARNING, ofpact_self_learning, ofpact).

**Figure 5:**
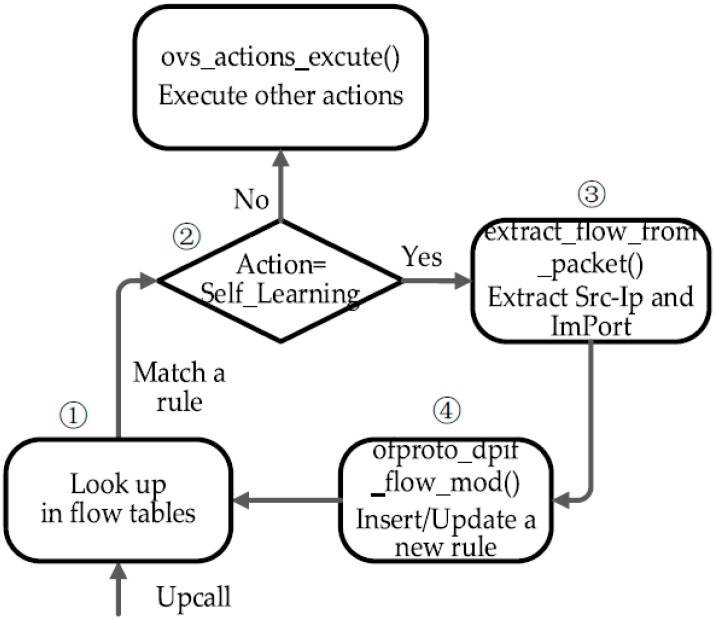
Self-learning module. Step 1 is to make packets match against rules in flow tables. Step 2 is to judge whether or not the rule’s action is Self_Learning. Step 3 is to extract Src-Ip and ImPort from the packet’s header. Step 4 is to insert/update a new rule according to the information extracted.

In addition to the above work, the flow chart (see in Figure 5) shows the remaining work for the process of Self_Learning module implementation. When a packet matches a rule containing the self-learning action, the corresponding action will be executed (see step 1 and step 2 in Figure 5). Therefore, a necessary function named xlate_self_learning_action should be defined in ofproto/ofproto-dpif-xlate.c. This function is to parse the packet and generate a new rule (see step 3 in Figure 5). Then it checks whether there is a rule as same as the new rule in the flow tables. If not, the new rule is to be inserted into the appropriate flow table. Otherwise, the old rule will be updated according to this new rule (see step 4 in Figure 5). Besides, we make some appropriate changes to some related functions in lib/ofp-actions.c file.

If a controller sends a Flow-Mod message containing a new match field and a new action to OVS. OVS can’t parse the new match field and the new action, so necessary modifications have to be made in some files to make them compatible with the new actions and match fields in the Flow_Mod message. These main files are lib/ofp-parse.c, lib/ofp-util.def, lib/match.c, meta-flow.c (struct mf_field), and nx_match.c.

## 4. Experimental Evaluation

In this section we describe our experimental evolution, including experimental setups, configurations and results. The goal of the experiments is to evaluate the feasibility of the proposed architecture when a SDN-based vehicle sensor network’s controller is unavailable. In SDN-based mobile agricultural networks, the node carrying a controller is easily disconnected from the sensor nodes carrying switches, so this experiment focuses on the controller failure recovery when the controller is unavailable. We use extended Ryu [23] as the controller and extended OVS (refer to 3) as an OpenFlow switch. The link between the controller node and switch nodes is wireless. Besides, there are some constraints in this testbed such as a variable radio signal level, limited processing power and limited memory [24]. According to some close research in this field [5,12,16], building this testbed simulates the real agricultural scenario adequately to evaluate the proposed architecture.

### 4.1. Configurations

In order to validate the proposed architecture and proof-of-concept prototype, we design a scenario about controller failure recovery in SDN-based vehicle sensor networks. When a controller connects to switches normally, all switches are controlled by the SDN controller, but the controller carried by a vehicle can disjoin the sensor network any time or the SDN-Apps of the controller can crash. In these scenarios, the controller is suddenly unavailable, and then the switches of sensor nodes will immediately handoff into disaster recovery mode. Switches will continue to execute the packet-processing logic installed by the controller proactively, which maintains the normal communication of a real vehicle sensor network in agriculture.

Controllers should install some appropriate rules into switches proactively. To describe the logic of design clearly, flow tables are multiple tables including three flow tables. As shown in Table 1, there are three flow tables. Flow table 0 is in charge of controlling the connection state, flow table 1 is used in normal connection scenarios and flow table 10 is used in failure recovery. Flow table 0 can implement the corresponding processing policy according to different connection states between the controller and switches. Flow table 1 performs the processing and forwarding of packets, and sending Packet-In messages to the controller when the controller is available. Flow table 10 stores some rules generated by self-learning actions. Besides, flow table 10 can ensure packet-processing and security control in the data plane when the controller is unavailable.

**Table 1:** Rules in the Proposed Architecture.

Flow Table	Priority	Entries
0	0	Match: connect_state = disconnection, Action = self-learning, go to table 10
	1	Macth: connect state = connection, Action = go to table 1
1	0	Match: any, Action = send to controller
	1	Rules installed by the controller when the controller is available
10	0	Match: any, Action = flood
	1	Rules generated by action Self-learning
	10	Secure rule: Match: dst_ip = 10.0.0.1, Action = drop

To understand this application/service about the controller failure recovery, we will describe how to process a packet in the flow table pipeline. In a sensor vehicle agricultural network, sensor data is transmitted by different sensor nodes. When a data packet arrives at a node’s OVS ingress port, the packet is first matched against a rule of flow table 0, which tells what to do according to different scenario states. If communication between the controller and the switch is normal, the rule’s action explicitly directs the packet to flow table 1. Flow table 1 will perform the processing of the packet, e.g., send the packet to the controller. If the controller node is unavailable and switches enter into disaster recovery mode, the packet can match the first rule of flow table 0, which executes action self-learning, generates a new rule and installs the new rule into flow table 10. Then the packet is sent to flow table 10 for further processing. For security purposes, the third rule in flow table 10 can drop the packet sent to a protected node who has a specific IP, such as 10.0.0.1. Otherwise, the packet can match against rules generated by self-learning action or the flood rule, which can ensure that communication is normal in the sensor network though the controller is suddenly unavailable.

Through building this proof-of-concept prototype of the disaster recovery service, it not only can ensure two different scenarios handoff fast and communication is normal, but also prevent the old rules from being used continuously when the network topology may change. To compare with our methodology, we introduce a traditional methodology [18] (an existing mechanism named fail-secure mode in OVS and OpenFlow protocol) into experiments whose flow table is shown in Table 2.

**Table 2:** Rules in traditional methodology.

Priority	Entries
0	Match: any, Action = logical port normal
1	Match: any; hard timeout = 1, Action = send to controller
10	Rules installed by the controller when the controller is available

The Hard_Timeout field of the second rule in the above flow table is set to be one second. This rule’s priority is higher than the first rule. The controller should maintain this rule at a regular basis, such as one second. Once the controller node is unavailable, packets will match the first rule and be processed by logical port Normal to maintain the network communication. This methodology is named B1s. The Hard_Timeout field of another methodology named B2s is set to be 2 s. B2s’s other configuration is the same as that of B1s. Under the controller failure recovery scenario, B1s and B2s are compared with the methodology of the proposed architecture.

### 4.2. Experiment Results

In this section, we describe the experiment results and analyze them from two points of view: (1) disaster recovery; (2) packet-processing performance.

#### 4.2.1. Disaster Recovery

**Figure 6:**
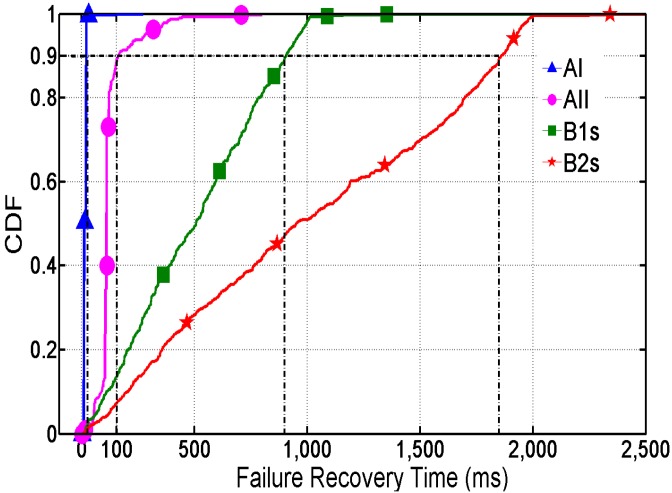
Failure Recovery Time of CDF.

We primarily focus on the performance of failure recovery and handoff from SDN controller connection loss. To make our methodology more convincing, we tested two SDN-based agricultural vehicle sensor network scenarios. When the SDN-Apps crash, the controller node will initiate to send a socket disconnection request to the sensor node switches, so the first scenario is that the controller service is down. The other scenario is that the mobile vehicle carrying a controller may suddenly disjoin the sensor network, which makes switches waste a lot of time to try to connect to the controller repeatedly, so the second scenario is that the wireless link between the controller and the switch is suddenly interrupted. The first scenario is named AI and the second one is named AII (refer to Section 2.2.1 about how to detect connection state in these two scenarios). Besides, we compare them with the traditional methodologies, such as B1s and B2s (refer to Section 4.1). We trigger the controller failure randomly and use different UDP throughputs to test. Figure 6 shows the failure recovery cumulative probability distribution by a series of tests.

In comparison with our methodology, B1s and B2s lead to a relatively long recovery time of the UDP session. The 90th percentiles are about 1 s in B1s and 2 s in B2s. In B1s and B2s, the controller should maintain the rule whose action is Controller on a regular basis (see the second rule in Table 2), which leads to a long handoff time (after the loss of communication with the SDN controller and before the rule expires). The recovery time will become longer with the growth of the rule’s Hard_Timeout. The long recovery time means that the sensor nodes can’t forward the received data packets to other nodes when the controller is unavailable.

On the contrary, with the our proposed methodology combining a connection-state processing module with a self-learning module, the result shows that communication restores fast after the controller becomes unavailable. In the first scenario, the 90th percentiles of recovery time is below 10 ms. The second scenario is about 100 ms. The second scenario is designed to disconnect the wireless link between the controller and the switch. Therefore, connection–state processing module can use finite state mechanism and probe mechanism to get real-time states like disconnection states in a short period. The result indicates that the state detection can be done quickly by the module. Therefore, the vehicle sensor network won’t suffer from more failure recovery delays.

**Figure 7:**
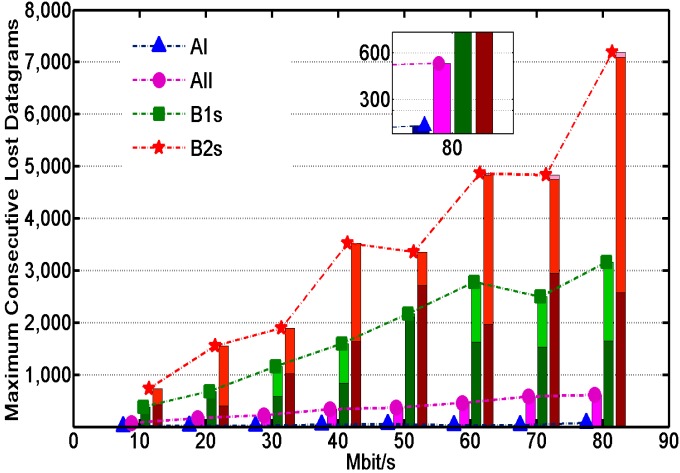
The number of consecutive packet losses during recovery time.

Figure 7 describes the number of consecutive sensor data packets lost during recovery time. The number of colors on each column represents the number of seconds of recovery time. The number of per Mbit UDP session is about eighty. Figure 7 indicates that the order of magnitude of total packet loss in B1s and B2s is higher than in AI and AII. The number of total packet losses increases proportionally and the loss time grows to 2 or even 3 s with the growth of throughput. Severe packet loss isn’t allowed in precision agricultural network. On the contrary, the loss time in AI and AII is below 1s. In the worst case, the number of packet losses in AI is below 10 and the packet loss rate is below 1 percent. The number of packet losses in AII is below 500 and the packet loss rate is below 8 percent, whose performance is still better than the traditional methodologies B1s and B2s.

**Figure 8:**
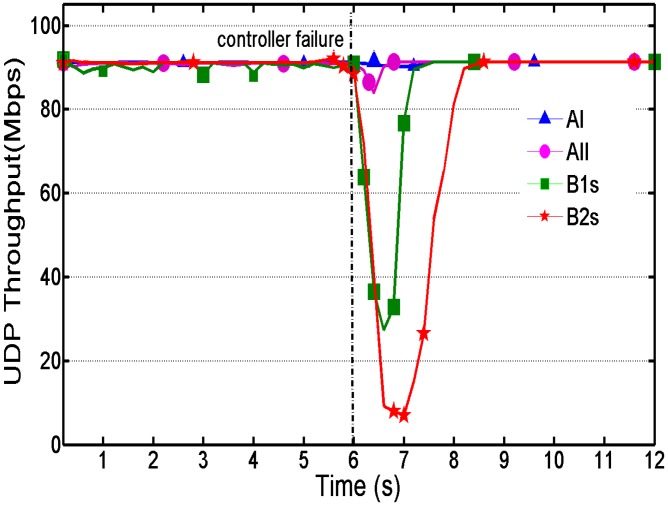
Instant throughput over time.

Figure 8 shows the instant UDP throughput over time, which can indicate how stable the vehicle sensor network is. Controller failure occurred 6 s after the beginning of the experiment.

As shown in Figure 8, the throughput of methodologies B1s and B2s is not stable before the controller failure occurs. This is because the controller has to install the specific rule in each switch and maintain the rule on a regular basis (see the second rule in Table 2), whose stability depends on quality changes of the wireless link, such as narrow-bandwidth or long latency in bad weather conditions. A more serious problem is the sharp decrease of throughput and relatively long recovery period when the controller is unavailable, which nearly leads to communication loss in the sensor network. On the contrary, the throughput of our proposed methodology stays stable constantly and the recovery time is so short that throughput only has a little fluctuation. When a controller reconnects to the switches, the handoff also has a good performance. These can ensure a stable sensor data transmission between different sensor nodes in agricultural networks.

#### 4.2.2. Basic Performance

As shown in Table 1, our proposed methodology needs to perform stateful matching, self-learning and forwarding. However, in Table 2, the compared traditional methodology only needs to execute the first rule whose action is Normal under the unavailable controller scenario. Besides, our proposed methodology is based on the designed architecture with extended OVS. The comparison traditional methodology is still based on the original OpenFlow Switch OVS. To prove whether the proposed architecture based on extended OVS maintains some of the basic performance of the original OVS, this section shows the basic performance features of our proposed methodology, such as RTT, throughput, CPU usage, *etc.* [20]. For convenience, the result of our proposed methodology is marked Approach-A. The result of the comparison traditional methodology is marked Approach-B. We used Netperf’s TCP_STREAM and UDP_STREAM to test TCP and UDP throughput [25]. Then under the same test conditions, we also tested round-trip time (RTT). The obtained results are presented in Table 3.

**Table 3:** The measured numbers of RTT Netperf TCP_STREAM and Netperf UDP_CRR.

Approach	RTT (ms)	TCP Throughput (Gbps)	UDP Throughput (Gbps)
Approach-A	0.41	2.91	6.24
Approach-B	0.43	2.92	6.12

As shown in Table 3, our proposed methodology’s performance is basically the same as the original OVS’s performance, which indicates that the speed of lookup and matching using our proposed connection state field is not reduced. The self-learning module is not only triggered flexibly by extended OpenFlow action, but also keeps as good throughput as the original OVS.

To further measure the packet processing performance using our proposed architecture, we use Netperf’s TCP_CRR and TCP_RR test. TCP_CRR repeatedly establishes a TCP connection, sends and receives one byte of traffic and disconnects. TCP_RR only establishes one TCP connection from the beginning. The results are reported in transactions per second (tps).

Table 4 shows that the number of TCP transmissions and CPU usage is kept in the same level as in the original OVS. In general, our architecture based on extended OVS won’t reduce the basic performance.

**Table 4:** The measured number of Netperf TCP_CRR, Netperf TCP_RR and CPU usage.

Approach	TCP RR (ktps)	TCP CRR (ktps)	CPU (%)
Approach-A	57.2	10.6	63.1
Approach-B	56.9	10.5	63.2

Therefore, the results from the above two parts show that our proposed methodology has relatively low failure recovery time, less packet loss and stable throughput. The measured number of throughput and TCP transmissions show our proposed architecture maintains the same basic performance of packet processing and forwarding as the original OVS. Therefore, these results show that the proposed architecture can improve the stability and survivability of a vehicle sensor network in agriculture.

More importantly, different connection scenarios in the agriculture field may have different vehicle sensor network topologies. In our methodology, packets can match against the corresponding rules in flow tables according to different scenario states, which can prevent the old rules from being used continuously and incorrectly, so in a SDN-based vehicle sensor network, what will not happen is that sensor data packets will be forwarded incorrectly because of the old rules. Besides, the flow table can drop specific packets, such as the packets with a specific IP or Port, to improve the security of the network when SDN controller communication is not reliable enough. This enhances the controllability of the vehicle sensor network under unavailable SDN controller conditions.

## 5. Related Work

Vehicle sensor networks have been extensively applied in precision agriculture, where they can increase efficiency, productivity and profitability in agricultural systems. A new sensor network architecture with autonomous robots [1] is proposed to monitor agricultural emergencies for farmers. A collaborative system made up of a wireless sensor network and an aerial robot [2] is applied to real-time frost monitoring in vineyards. Sensors and GPS are used to improve the accuracy of vehicle positioning in precision architecture [5].

Introducing SDN to vehicular sensor networks in agriculture could improve the management of mobile network resources [14] and increase the production efficiency. However, when a SDN controller carried by mobile vehicle cannot communicate with switches on other sensor nodes, the performance of flow-based forwarding of switches will be degraded. The attempts to resolve the problem are as follows:

Failure recovery in the data plane: Failure recovery and reliability are fundamental requirements of SDNs, including SDN-based vehicular sensor networks in agriculture. A new language named FatTire [26] and a fast restoration mechanism in OpenFlow [27] are proposed to realize connectivity recovery for link failures in the data plane. LegoSDN [28] is proposed to protect SDN-Apps against failure, as opposed to complete controller crash failures. In contrast, this paper proposes a methodology to achieve network recovery quickly when controller communication with switches isn’t available and switches are out of control.

Optimizing the handoff mechanism: optimizing the election mechanism for selecting a second controller as a new main controller when the main controller is unavailable [16]. The time of selection and handoff will be longer with the growth of the number of network nodes. In this interval of selection, switches will continue to use the old rules and can’t process the new packets because of loss of control. The Optimizing Ravana Protocol [17] makes a slave controller to take over fast when the master controller crashes. However, once a wireless link is not stable, it becomes hard to connect other controllers, in which case the recovery time will be longer. In this paper, the experimental results demonstrate that communication can remain normal though all of controllers are unavailable.

Using middleboxs or a local agent [12]: Pulsing a local agent in the data plane controls the network instead of a controller when a controller is unavailable, but the experimental results show that throughput will down by 40 percent after a controller disconnects. Moreover, local agents limit the flexibility and benefits of SDN. The architecture proposed in this paper has stable throughput and maintains the benefits of SDN by extended OpenFlow.

Using the double pipeline of OpenFlow: OpenFlow specification [18] and some switches (e.g., Open vSwitch [22]) support logical port such as Normal. A packet enters the OpenFlow pipeline and then can be processed as traditional L2/L3 functions of switches through the logical port Normal, which can ensure that the switch processes and forwards packets when a controller disconnects in SDN-based vehicular sensor networks. In fail-secure mode, it needs the controller to keep rules on regular time to ensure the controller works well, which leads to longer failure recovery times with the growth of the rule’s expiration time. In fail-standalone mode, after a controller disconnects, the switches will allow all traffic to forward, which easily causes security problems. In addition, the compatibility of the Normal port and other ports is poor.

## 6. Conclusions

Nowadays, SDN-based vehicular sensor networks are becoming more and more popular in the precision agriculture field. The introduction of a centralized controller makes the vehicle sensor network data plane simple and efficient. However, the unstable wireless links in mobile scenarios may lead to connection failures between the controller and switches. Hence the vehicular sensor network has to select a main controller again, and communication will be interrupted because switches do not know how to process and forward new packets because of the loss of control. Meanwhile, the topology of vehicular sensor networks in agriculture can easily change and then the old rules continue to be used incorrectly. According to the idea of stateful matching and a packet-processing service invoked in the data plane, this paper proposes a SDN-based vehicular sensor network architecture based on extended OVS.

The proposed architecture mainly develops a connection-state processing service and self-learning service. We also attempt to make the design modular and implement it in extended OVS. The connection-state processing service can track the connection state between a controller and switches. Then it can synchronously transmit the state to the kernel datapath and user space and then it performs stateful matching. This mechanism can achieve smooth handoffs as the scenario state changes and maintains throughput stable in vehicle sensor networks. The self-learning service can enhance packet-processing capability in the data plane, such as L2/L3 functions. If so, the switch can decrease the number of Packet-In packets sent up to controller, which avoids some packet-proceeding delays and reduces the controller load. Of course, this architecture can ensure that a packet is processed and forwarded properly after the loss of communication with the SDN controller. Besides, sensor data packets can match against the corresponding stateful rules in different scenarios, which can prevents rules from being used incorrectly when the vehicle sensor network topology changes.

The experimental results show that, compared with traditional solutions (refer to Section 4), the solution we propose can decrease the effect of controller failure and strengthen the stability and survivability of SDN-based vehicular sensor networks in the precision agricultural field. More importantly, it is worth considering that the ideas of stateful matching and a packet-processing service called in the data plane can be applied in more agricultural scenarios. 
